# The Effect of Intraset Rest Periods on External and Internal Load During Small-Sided Games in Soccer

**DOI:** 10.3390/sports12120331

**Published:** 2024-12-02

**Authors:** Ioannis Ispirlidis, Dimitrios Pantazis, Athanasios Poulios, Alexandra Avloniti, Theodoros Stampoulis, Yiannis Michailidis, Konstantinos Troupkos, Evangelos Evangelou, Dimitrios Draganidis, Dimitrios Balampanos, Nikolaos-Orestis Retzepis, Maria Protopapa, Nikolaos Mantzouranis, Nikolaos Zaras, Maria Michalopoulou, Ioannis G. Fatouros, Athanasios Chatzinikolaou

**Affiliations:** 1 School of Physical Education, Sport Science and Occupational Therapy, Democritus University of Thrace, 69100 Komotini, Greece; iispyrli@phyed.duth.gr (I.I.); dpantazi@phyed.duth.gr (D.P.); alavloni@phyed.duth.gr (A.A.); tstampou@phyed.duth.gr (T.S.); kostastroupkos5678@gmail.com (K.T.); baggelis.euaggelou10@gmail.com (E.E.); dimibala10@phyed.duth.gr (D.B.); nretzepi@phyed.duth.gr (N.-O.R.); mprotopa@phyed.duth.gr (M.P.); nmantzou@phyed.duth.gr (N.M.); nzaras@phyed.duth.gr (N.Z.); michal@phyed.duth.gr (M.M.); achatzin@phyed.duth.gr (A.C.); 2 Schools of Physical Education, Sport Science and Dietetics, Department of Physical Education and Sport Science, University of Thessaly, 42100 Trikala, Greece; apoulios@uth.gr (A.P.); ddraganidis@pe.uth.gr (D.D.); ifatouros@uth.gr (I.G.F.); 3 Laboratory of Evaluation of Human Biological Performance, New Buildings of Laboratories, Department of Physical Education and Sports Sciences, Aristotle University of Thessaloniki, University Campus of Thermi, 57001 Thessaloniki, Greece; 4 Department of Life Sciences, School of Life and Health Sciences, University of Nicosia, 2417 Nicosia, Cyprus

**Keywords:** external load, internal load, recovery period, interval training, soccer training

## Abstract

The purpose of this study was to compare the internal and external load in continuous and intermittent small-sided games (SSG) formats. Eight semi-professional soccer players participated in the study, and they completed three protocols: (a) I-intermittent SSG protocol (Int-I, 4 sets of 4 min with a 3 min recovery); (b) Continuous SSG protocol (Con, 2 sets of 8 min with a 3 min recovery); (c) II-SSG protocol (Int-II, 4 sets of 4 min, where each set includes 1 min of exercise with varying recovery periods (10, 20, 30 s), with a 3 min recovery period between sets). A one-way analysis of variance (ANOVA) was used to analyze the dependent variables, with significance determined at *p* < 0.05. The three protocols differed in total distance covered and in distance covered at speeds >19 km/h, with the Int-II protocol resulting in the greatest distance covered (*p* < 0.05). Additionally, players in the Con protocol exercised at a higher percentage of their maximum heart rate (%HRmax) (*p* < 0.05), while the highest RPE value was observed in the Int-I interval protocol (*p* < 0.05). The external load experienced by players in intermittent SSG protocols is higher, while internal load (%HRmax) remains relatively low. This effect is especially notable in the new intermittent exercise model proposed in this study, which incorporates progressively increasing recovery times within each exercise set. Coaches can use this model to increase players’ external load without causing a heightened perception of fatigue.

## 1. Introduction

The fundamentals of soccer match play include a variety of technical skills combined with multidirectional physical activity [1,2]. Due to their high degree of individualization and the likelihood of changing every 4–6 s, these activities become even more complex [3,4]. Soccer coaches, regardless of level, frequently employ small-sided games (SSG) to help players improve their technical and tactical skills as well as their physical conditioning [5,6,7]. Variations in the amount of time spent engaging in high-intensity activity, the distance traveled, and the repeatability of SSGs are caused by the size of the field, the number of participants, the game regulations, and coach encouragement [8]. Consequently, it is believed that these variables cause variations in heart rates (HRs), blood lactate (ΒL), and the percentage of maximal HR (HRmax) [9,10].

Nowadays, locomotor activity data can be collected more quickly and in greater amounts due to the development of Global Positioning System (GPS) technology, which adds to the amount of data available about SSG responses [11]. Commercially accessible GPS receivers have previously been proven to be valid and reliable, which has allowed game-related conditioning exercises to be more accurate [12]. As a physical conditioning technique, small-sided games can elicit heart rate responses that are between 90 and 95% of maximum heart rate (HRmax) [13,14]. Chronic exposure to these levels of intensity, when provided through small-sided games, improves physical and aerobic fitness [5].

Pitch size and number of players are two of the many variables that affect soccer’s SSGs [5,9]. With players covering longer distances overall and running intensely for longer periods of time, larger pitches typically result in higher physical demands [15]. Specifically, it has been observed that the two SSG types (small, 20 × 25m; large, 70 × 65 m) were comparable in respect to total distance, mean speed, and intense accelerations and decelerations. In the large SSG, players had higher high-intensity running, high-speed running and maximum speed than in the small SSG [9]. On the contrary, the mean HR, peak HR, and time at >90% of HRmax was greater in the small SSG compared to the large SSG [9]. Additionally, smaller formats, specifically 2v2 and 3v3 games, elicit higher HR and ratings of perceived exertion (RPE) compared to larger formats such as 4v4 and 5v5 [12]. In these smaller formats, players experience HR responses that approach 90% of their HRmax, indicating a higher level of physiological stress during gameplay [12,16]. Players’ activity profiles vary among SSG formats, with the 3v3 format covering the longest distance at high speeds (18 km/h or more), while the 2v2 format results in the lowest distance covered and fewest sprints [12]. This variation in activity profiles highlights the importance of selecting the appropriate SSG format to achieve specific training outcomes, whether they be endurance or speed.

Despite the fact that 4v4 SSGs seem to be a good method to increase power-related soccer actions and simulate game conditions [17], the pitch size and the player’s number are only a part of the training load determination via SSGs [18]. It has been observed that 4v4 SSGs with 4 sets, each lasting four minutes, promote cardiovascular adaptations and increase the mean HR, the speed, and the RPE levels of soccer players [19]. Furthermore, it appears that using a different set-to-set time ratio (6 min × 2 sets) limited HR levels and game intensity while increasing fatigue [20]. Overall, it is considered expected that the set duration influences players’ physiological responses [21]. Specifically, lower set durations (4 min) could induce higher RPE levels compared to longer set durations (6 min) [22]. However, the HR responses may not differ meaningfully between 2 and 4 min set durations [23], highlighting the importance of implementing a recovery period between sets [24].

The recovery period between sets can vary. The effects of short (30 s–1 min), medium (1.5–2 min), and long (3–4 min) recovery periods have been examined [24,25], indicating that the longer recovery period contributes to a complete recovery and higher performance level in subsequent bouts [24]. Additionally, the recovery times of 30 s and 120 s were compared, but no difference was observed in external load indicators [24]. However, the HR responses were higher following the 30 s recovery period [24]. It seems that the differentiation of work-to-recovery ratios in SSGs could influence RPE levels but could not have a meaningful effect on performance indicators [26]. Furthermore, the versatility of SSG structures in soccer training and the lack of comparison of different work-to-recovery ratios induced the need for investigation of SSGs with different structures [18,24,26]. Although there are studies that examine how soccer players’ workloads vary depending on the distance and number of players during SSGs, data have not been investigated in correlation to the work-to-rest ratio. Therefore, the aim of this study was to examine the acute physiological responses and external and internal load of three soccer SSG (4v4) formats (one continuous protocol and two intermittent ones) while maintaining the pitch size. It is considered that this investigation will clarify the effect of different work-to-recovery periods on soccer performance indicators, contributing to the appropriate selection of the structure of SSGs.

## 2. Materials and Methods

### 2.1. Participants

A power analysis (G*Power, version 3.1.9.2) indicated that eight participants would be an appropriate sample size (power = 0.80, effect size = 0.3, probability error = 0.05) [27,28]. The eight participants were semi-professional soccer players. The semi-professional players participated in an average of five practices and one game per week during the season, depending on the competition schedule. Additionally, the soccer players were performing high-intensity interval training twice a week for 20 min in each practice. For inclusion, the following criteria were used: (a) no recent medical history of illness or injury; (b) no use of ergogenic supplements or medications; and (c) no use of alcohol or tobacco during the three months prior to the study’s beginning. The characteristics of the study participants are shown in Table 1. Following an extensive description of the study’s aims, methods, risks, and benefits, the volunteers answered a series of medical questions and signed a written consent form.

The 2013 version of the Helsinki Declaration was followed. The study was approved by the Democritus University of Thrace’s institutional ethics committee (29660/206 Date 21 January 2022).

### 2.2. Study Overview

**Figure 1:**
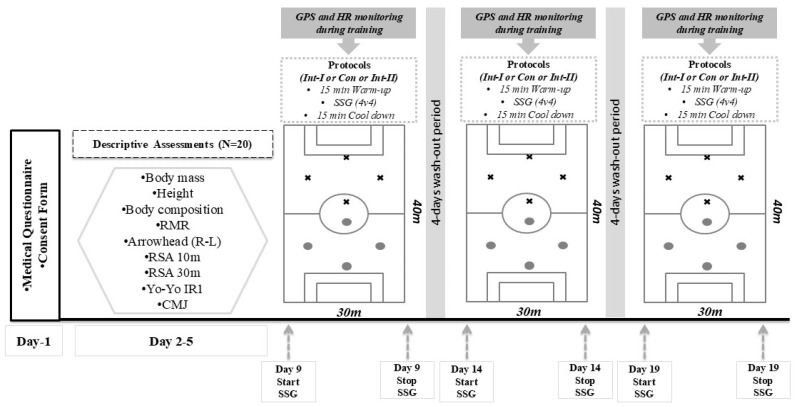
Experimental Design. RMR, resting metabolic rate; RSA, repeated sprint ability; Yo-Yo IR1, Yo-Yo intermittent recovery test level 1; CMJ, counter movement jump; R, right; L, left; m, meter; SSG, small-sided games; Int-I, intermittent-I protocol; Con, continuous protocol; Int-II, intermittent-II protocol; GPS, global positioning system; HR, heart rate.

A randomized, three-trial crossover design was implemented (Figure 1). In order to limit detraining and ensure that players were still used to rigorous conditioning and game load, the study was performed one week following the in-season. Volunteers participated in three protocols: (a) I-intermittent SSG protocol (Int-I); (b) Continuous SSG protocol (Con); and (c) II-SSG protocol (Int-II). Participant body mass, height, body composition, and performance were measured prior to the first protocol execution, and all procedures were explained to them. All the SSG protocols were performed on a natural grass field under the same conditions (20–22 °C). In between trials, a 4-day washout period, during which soccer players participated in daily light training, was applied between protocols. This 4 day period was preferred, since it has been observed that 48 h after SSG execution, 72 h is a sufficient time for the recovery of semi-professional soccer payers [29]. Heart rate monitoring and global positioning system (GPS) devices were used to measure field locomotor activity during protocols. Soccer players were told to keep to their usual diets during the trials.

### 2.3. SSG Protocols

The first SSG protocol consisted of 4 sets of 4 min, with 3 min recovery periods between sets (Int-I). The second SSG protocol consisted of 2 sets of 8 min, with 3 min recovery periods between sets (Con). Additionally, the third SSG protocol consisted of 4 sets of 4 min, with the structure of each set including 1 min of exercise and recovery periods of various durations (10″, 20″, 30″) and a last minute of SSG, including a 3 min recovery period between sets (Int-II). The 4v4 SSG condition appears to be an effective training method for increasing power-related soccer actions and simulating game demands [17]. Furthermore, the SSGs structured as four sets of four minutes for each set enhanced physiological responses and technical ability in soccer players, increasing heart rates and promoting cardiovascular levels [19]. Additionally, in longer continuous SSGs (6–12 min), a restricted intensity in the second set has been observed [20]. Although the duration of the recovery time was the same across all trials, the method of administration varied. The short (30 s–1 min) and medium (1.5–2 min) recovery periods are used to balance the training load inducing high internal and external load responses [25], and the long recovery period (3–4 min) is used to allow for more complete recovery and a higher performance level in subsequent bouts [24]. The warm-up was standard for all protocols (15 min, shuttle running, dynamic stretching, passing exercise, and agility drills with and without ball). After each protocol execution, a standard 15 min cool-down was performed. The SSG (4V4) field size was the same across all protocols (40 × 30 m). Players were encouraged to observe SSG rules (score a goal after completing 6 passes, man-to man marking, no goalposts) by the same soccer coach.

### 2.4. Measurements

#### 2.4.1. Descriptives

Body mass and height were measured on a beam balance using a stadiometer (Beam Balance-Stadiometer; SECA, Vogel and Halke, Hamburg, Germany). Dual-energy X-ray absorptiometry (Lunar DPX-NT; GE Healthcare, Madison, WI, USA) was used to assess the body’s composition. Soccer-specific performance was evaluated using a Yo-Yo intermittent recovery level 1 test, as has been described previously [11]. Countermovement jump (CMJ) was measured using an optical measurement system consisting of a transmitting and receiving bar (EzeJump, Swift Performance, Lismore, Australia) [11]. Repeated sprint ability (RSA, 5 × 10.30 m) was measured with an accuracy of 0.01 s using infrared photocells (DUO™ Timing Gates (Swift Performance, Lismore, Australia) [30]. The arrowhead agility drill test was used to evaluate the ability of changes in direction with an accuracy of 0.01 s via infrared photocells (DUO™ Timing Gates (Swift Performance, Lismore, Australia) [31]. Following an overnight fast, an open-circuit canopy unit system (Vmax Encore 29, BEBJO296, Yorba Linda, CA, USA) and a calculation technique were used to determine the resting metabolic rate (RMR) [11].

#### 2.4.2. Field Activity

Field activity and internal load during protocols (Int-I, Con, Int-II) were monitored using a high time resolution global positioning system equipped with heart rate monitors (10 Hz GPS, 200 Hz triaxial accelerometry; Polar Team Pro, Polar Electro, Kempele, Finland), as previously described [30]. Activity was classified as total distance; distance zone 1 (Dist zone 1, distance covered at speed 3–6.99 km/h); distance zone 2 (Dist zone 2, distance covered at speed 7–10.99 km/h); distance zone 3 (Dist zone 3, distance covered at speed 11–14.99 km/h); distance zone 4 (Dist zone 4, distance covered at speed 15–18.99 km/h); distance zone 5 (Dist zone 5, distance covered at speed > 19 km/h); number of accelerations (2 to 2.99 and >3 m/s^2^); and number of decelerations (−2 to −2.99 and >−3 m/s^2^). The average heart rate (as a percentage of HRmax) and the classification of heart rate as time in HR Zone 1 (50–59% of HRmax); time in HR Zone 2 (60–69% of HRmax); time in HR Zone 3 (70–79% of HRmax); and time in HR Zone 4 (80–89% of HRmax) were measured during protocols. The 1–10 Borg scale was used to assess the rate of perceived exertion (RPE) and the sum rate of perceived exertion (sRPE) [32].

### 2.5. Statistical Analysis

Data are presented as mean ± SD. The normality check for all dependent variables was examined via the Shapiro–Wilk test (a non-parametric test was not necessary). A one-way analysis of variance (three levels) was used to analyze the dependent variables. The Bonferroni’s multiple comparison test (for pairwise comparison) was used when a statistically meaningful effect was detected. Significance was determined at *p* < 0.05.

## 3. Results

From the statistical analysis, significant differences were observed between the three protocols concerning the TD parameter and the distance covered at a speed of 3–6.99 km/h, with players covering the shortest distance under the Con protocol. Additionally, differences were found in the distance covered at speeds > 19 km/h, where players in the Int II protocol covered the greatest distance compared to the other two protocols. In the Con protocol, players also covered a greater distance compared to the Int I protocol. No differences were observed between the protocols for the remaining variables. The mean values and statistical indicators of the variables are presented in Table 2.

Regarding internal load, the statistical analysis showed that players in the Con protocol exercised at a higher %HRmax and with a higher average HR compared to the two interval protocols. Analyzing exercise time within different HR zones revealed that the time in the Con protocol was significantly shorter compared to the other two protocols in the 60–69% HRmax, 70–79% HRmax, and 80–89% HRmax zones. Furthermore, the RPE analysis indicated that the Int I interval protocol yielded the highest value compared to the other two protocols. In addition, for the sRPE variable, the Con protocol showed a significantly lower value than the other two protocols, with the two interval protocols also differing; Int I displayed the highest value. The mean values and statistical indicators are detailed in Table 1.

## 4. Discussion

In this study, three different SSG exercise protocols were used: one continuous protocol (CON) and two intermittent ones (Int I and Int II). It is worth highlighting that in each four min set of Int II, three minutes were spent on exercise and one on recovery. Thus, the total net exercise time in Int II was 12 min, unlike in the other protocols, where it was 16 min. Despite the reduced time, players exhibited higher external load on most variables, indicating that the brief intermittent recovery periods helped maintain a high pace, likely facilitating phosphocreatine resynthesis and the removal of metabolic by-products [33,34].

Starting with the total distance, players covered 23% more distance with Int II than with Con and 12% more than with Int I. Additionally, in Int I, they covered 12% more distance than in Con. Similar findings were reported in previous studies, such as in a study by Hill-Haas et al. (2009), who observed that fractionated approaches allowed players to cover more distance than continuous methods [35]. Likewise, Branquinho et al. (2020) found that players covered greater distances in two fractionated protocols compared to a continuous one [36]. Studies by Clemente et al. (2018, 2019) also observed that players covered more distance in a protocol with six 3 min sets compared to a protocol with three 6 min sets [37,38]. These results align with other studies like that by Koklu et al. (2017), which noted that total distance (TD) was higher in protocols with more sets than in continuous ones [39].

Regarding distances covered at different speeds, this study found differences at the 3–6.99 km/h speed, where the Con protocol showed the shortest distance compared to the other protocols. Additionally, Int II exhibited the greatest distance at this speed range. Players covered less distance in the Con protocol at speeds of 7–10.99 km/h, while in Int II, they covered the most distance at speeds of 11–14.99 km/h and >19 km/h. Similar results were reported in studies like that conducted by Casamichana et al. (2013), who noted that, in intermittent protocols, players covered more distance at speeds under 6.9 km/h [40]. However, it should be noted that the speed zones defined in those studies differ from those in the current study. Koklu et al. (2017) reported that the greatest distance while walking was observed in the continuous protocol, while in protocols with multiple sets of short durations (6 × 2′), players covered more distance at all other movement speeds (7–12.9 km/h, 13–17.9 km/h, >18 km/h) [39].

In the current study, the only differences observed were in decelerations (<−3 m/s^2^), while no differences were noted in accelerations across the three protocols. Clemente et al. (2019) found that the protocol with the most sets (6 × 3′) had 24% more accelerations and 26.7% more decelerations compared to the protocol with fewer sets (3 × 6′) [37].

Before proceeding, it should be noted that one parameter significantly influencing both external and internal load is the field size used for each SSG protocol and the player-per-square-meter ratio resulting from this. The present study used a 40 × 30 m field, equating to 150 m^2^ per player. Only Hill-Haas et al. (2009) used a similar ratio, making comparison with their findings more accurate [35]. Branquinho et al. (2020) used a 5v5 format with a ratio of 160 m^2^ per player [36]. Other studies, such as Koklu et al. (2017), used a 4v4 format on a 32 × 25 m field (100 m^2^ per player), while Clemente et al. (2019) and Casamichana et al. (2013) used 5v5 formats on fields measuring 42 × 22 m (92 m^2^ per player) and 55 × 38 m (209 m^2^ per player), respectively [37,40]. Therefore, when comparing findings across studies, this factor should be considered, as the player-per-square-meter ratio affects the external load [41,42].

All these variables reflect the external load experienced by players across the three protocols. The body’s response to external load constitutes the internal load. In this study, heart rate (HR) and RPE (Rating of Perceived Exertion) were observed. Beginning with the mean HR across the three protocols, it was noted that HR was 5.3% and 7% higher in Con compared to Int I and Int II, respectively. Players in the Con protocol exercised at the highest percentage of HRmax (88% HRmax). Similar findings were observed by Hill-Haas et al. (2009), who reported that continuous SSGs led to a higher %HRmax than intermittent SSGs (87% vs. 84% HRmax) [35]. This trend was also found by Koklu et al. (2017) in various formats (4v4, 3v3, and 2v2) [39]. However, a recent study conducted on elite youth players found no differences in HRmax percentage across three protocols (8 min, 2 × 4′, 4 × 2′). This study used a 6v6 format on a 50 × 32 m field (133 m^2^ per player) [26]. A lack of differences between continuous and intermittent protocols was also reported by Casamichana et al. (2013), where %HRmax was consistently high (>87%) across all protocols [40].

The differences observed in this study are likely due to the rest intervals in the two Int protocols, allowing HR reduction from set to set, so each set begins from a lower HR, resulting in a lower overall HR than in the Con protocol. Differences in findings across studies may be due to variations in participant skill level, exercise-to-rest ratio, player-per-square-meter ratio, and design (total exercise duration).

Regarding the time players spent in different HR zones, differences were observed only for the 60–69% HRmax, 70–79% HRmax, and 80–89% HRmax zones, with Con having the shortest times. No significant differences were observed in the 90–100% HRmax zone, though the longest times were noted in Int I. It appears that players in Int II spent the most time in intensities above 80% HRmax compared to the other protocols. Only one study with a similar design (comparison of continuous and intermittent SSGs) was found in the literature, reporting that 60–70% of total exercise time was spent at >84% HRmax [40].

In each set of Int I, mean HR increased progressively, with significant differences observed between the first and fourth sets (10% increase), while no changes were seen in the other two protocols. Casamichana et al. (2013) also reported that %HRmax was lower in the third and fourth four-minute periods of the continuous method, while in the intermittent protocol, the lowest %HRmax was noted in the first set (0–4 min) [40]. Similar trends were observed in another study [23], where lower values were noted in the first four-minute set compared to the following three sets.

As previously mentioned, Int I exhibited the highest RPE values, significantly differing from those in the other two protocols. This observation aligns with previous findings [43]. Likewise, a similar difference was observed for the sRPE variable in the current study. Contrarily, several studies reported the highest RPE values following continuous SSG protocols [26,35,39]. Clemente observed in two studies (2018, 2019) that RPE increased with set duration (higher RPE in the 3 × 6′ protocol compared to the 6 × 3′ protocol) [37,38]. The observed difference in this study could partly be attributed to the external load recorded, as lower TD in Con may have influenced RPE.

It should be noted that the external and internal load of a program, especially when it takes the form of SSGs, depends on factors such as the gender, level, and current physical condition of the athletes. Notably, the same game is played with different intensity levels when the level of the football players changes. As previously mentioned, thisstudy involved male semi-professional football players whose physical condition, based on their performance in the Yo-Yo Intermittent Recovery Level 1 test, can be characterized as low. Therefore, our findings should be interpreted in consideration of the sample’s characteristics.

This study also presents certain limitations. Furthermore, even if there are enough participants for statistical analysis, the study’s restricted sample size might be seen as a limitation because more participants could facilitate data generalization. Additionally, one limitation is that only male semi-professional football players were included. Finally, the use of specific SSGs (structure, duration, rest intervals, player ratios) constitutes yet another limitation. Therefore, further studies with larger samples of different levels, ages, and genders are needed to draw more reliable conclusions regarding the impact of these programs.

## 5. Conclusions

The external load experienced by players in intermittent SSG protocols is higher, while the internal load (%HRmax) remains relatively low. This observation is particularly pronounced in the new intermittent exercise model proposed in this study, which incorporates progressively increasing recovery time within each exercise set. In the training process, this SSG model could be used by fitness coaches to load football players to higher external loads with a lower perceived sense of fatigue. Additionally, the short recovery intervals during the sets can be utilized by coaches to provide quality (technical-tactical) feedback to the players.

## Figures and Tables

**Table 1:** Participants’ characteristics.

Age (years)	21.47 ± 0.29
Height (m)	1.78 ± 0.06
Body mass (kg)	73.86 ± 7.94
Training Age (years)	15.00 ± 2.83
Body Fat (%)	15.71 ± 3.73
RMR	1967.50 ± 260.80
Arrowhead Right (s)	8. 50 ± 0.21
Arrowhead Left (s)	8.69 ± 0.22
RSA 10 m (s)	1.78 ± 0.05
RSA 30 m (s)	4.39 ± 0.14
Yo-Yo IR1 (m)	1.290 ± 359.52
Yo-Yo HRmax (b/min)	189.00 ± 6.68
CMJ (cm)	34.53 ± 2.43

RSA, repeated sprint ability; RMR, resting metabolic rate; Yo-Yo IR1, Yo-Yo intermittent recovery level 1; Yo-Yo HRmax; maximal heart rate; CMJ, counter movement jump.

**Table 2:** Mean values (MV ± SD) and statistical indicators of the study variables.

GPS Variables	Int-I	Con	Int-II	F	*p*
Heart Rate Average (%HRmax)	82.6 ± 54.96 *	87.88 ± 6.49	83.13 ± 3.64 *	5.333	0.019
Time in HR Zone 1 (50–59% HRmax) (s)	94.88 ± 128.64	19.63 ± 32.71	78.25 ± 51.35	3.426	0.061
Time in HR Zone 2 (60–69% HRmax) (s)	319 ± 181.35 *	127.87 ± 81.55	297.88 ± 122.08 *	6.874	0.008
Time in HR Zone 3 (70–79% HRmax) (s)	408.38 ± 142.67 *	120.37 ± 73.30	270.63 ± 79.39 *^,#^	15.069	<0.001
Time in HR Zone 4 (80–89% HRmax) (s)	420.25 ± 167.59 *	173.00 ± 119.97	591.50 ± 325.21^*^	12.152	0.001
Time in HR Zone 5 (90–100% HRmax) (s)	708.50 ± 258.38	685.75 ± 271.62	665.63 ± 408.74	0.081	0.923
RPE (AU)	8.63 ± 1.06 *	6.88 ± 1.13	7.50 ± 1.07 ^#^	10.897	0.001
sRPE (AU)	241.50 ± 29.70 *	130.63 ± 21.40	187.50 ± 26.73 *^,#^	70.456	<0.001
Total Distance (m)	2351.7 ± 127.87 *	2055.3 ± 132.10 *	2673.4 ± 168.46 *^,#^	31.373	<0.001
Dist zone 1 (3–6.99 km/h) (m)	609.38 ± 61.09 *	562.75 ± 63.89	805.25 ± 138.41 *^,#^	25.569	<0.001
Dist zone 2 (7–10.99 km/h) (m)	724.38 ± 138.44 *	586.75 ± 113.23	665.13 ± 110.01 *	5.552	0.017
Dist zone 3 (11–14.99 km/h) (m)	599.25 ± 56.70	553.25 ± 100.38	668.25 ± 70.68 *^,#^	6.077	0.013
Dist zone 4 (15–18.99 km/h) (m)	198.75 ± 72.93	224.38 ± 97.00	282.25 ± 106.51	3.040	0.080
Dist zone 5 (>19 km/h) (m)	23.38 ± 13.32	41.00 ± 24.27	73.50 ± 43.53 *^,#^	8.513	0.004
Dec (−2 to −2.99 m/s^2^) (n)	33.25 ± 9.39	31.38 ± 8.75	34.88 ± 10.09	0.426	0.661
Dec (<−3 m/s^2^) (n)	4.38 ± 3.42 *	10.75 ± 4.86	10.75 ± 7.85^#^	5.539	0.017
Acc (2 to 2.99 m/s^2^) (n)	26.63 ± 10.08	29.13 ± 9.22	34.75 ± 11.83	1.576	0.241
Acc (>3 m/s^2^) (n)	3.00 ± 2.33	4.00 ± 1.41	4.63 ± 2.56	1.396	0.280

Con *, differences between int-I and Con; Int-I ^#^, Differences between Int-I and Int-II; Int-II 3 *: Differences between con and Int-II.

## Data Availability

The data presented in this study is available on request from the corresponding author. The data are not publicly available due to research rules.
